# Structural analysis of the flexibility of the Ubl2 domain within the papain-like protease of SARS-CoV-2

**DOI:** 10.1107/S2053230X26003699

**Published:** 2026-05-18

**Authors:** Gian Luca Freiherr von Scholley, Martina Schaefer, Mark D. Tully, Marc-Andre Hograindleur, Montserrat Soler-López, Roman C. Hillig, Christoph Mueller-Dieckmann, Eaazhisai Kandiah

**Affiliations:** a European Synchrotron Radiation Facility (ESRF) 71 Avenue des Martyrs 38000Grenoble France; b Nuvisan (Germany) Muellerstrasse 178 13353Berlin Germany; University of São Paulo, Brazil

**Keywords:** SARS-CoV-2, papain-like proteases, Ubl2 domain, COVID-19, zinc binding, flexibility, NSP3

## Abstract

The papain-like protease (PLpro) of SARS-CoV-2 is part of the multi-domain nonstructural protein 3 (NSP3) and consists of two domains: a ubiquitin-like domain 2 (Ubl2) and a protease domain. PLpro plays a crucial role in the replication cycle of SARS-CoV-2, facilitating host immune-system evasion and the formation of double-membrane vesicles where replication occurs. While the function of the Ubl2 domain is still not clear, it is critical for the stability and the functional efficiency of PLpro. Despite its predicted inherent flexibility, nearly all SARS-CoV-1 and SARS-CoV-2 PLpro crystal structures deposited in the Protein Data Bank show the Ubl2 domain in a highly similar, closed conformation against the catalytic domain. Here, we present a crystal structure of PLpro exhibiting Ubl2 in two distinct conformations: the well characterized closed state, where Ubl2 is positioned near the PLpro domain, and an as yet uncharacterized open state, where Ubl2 is displaced by 4 Å from the PLpro core. This conformational variability in our structure appears to be related to the occupancy of a zinc ion within the zinc-finger domain. These results provide new insights into the flexibility of Ubl2, suggesting potential avenues for targeting and harnessing this dynamic behaviour for drug discovery.

## Abstract 2

The ubiquitin-like domain 2 (Ubl2) of the SARS-CoV-2 virus is necessary for the stability and catalytic efficiency of its papain-like protease (PLpro). Our crystallographic study reveals that the Ubl2 domain exhibits notable flexibility and can adopt a conformation that places itself away from the PLpro catalytic domain, representing a new conformation from those reported so far for SARS-CoV-1 or SARS-CoV-2. The structural flexibility of Ubl2 could be allosterically related to the stability of the zinc-finger domain.

## 1. Introduction

Severe acute respiratory syndrome coronavirus 2 (SARS-CoV-2), the virus responsible for the COVID-19 pandemic, has infected over 770 million people and caused seven million deaths from its first occurrence in 2019 until March 2026 (World Health Organization, 2026 ▸). While emerging variants challenge the effectiveness of existing vaccines and antiviral drugs such as Paxlovid, remdesivir and molnupiravir, they also indicate an urgent need for the discovery of new effective therapeutics, particularly for combinatorial treatments (Chan *et al.*, 2024 ▸). Addressing this challenge will require both *de novo* antiviral drug discovery and systematic drug-repurposing strategies (Du *et al.*, 2026 ▸; Oktavianawati *et al.*, 2023 ▸). However, the development of novel therapies relies on a thorough understanding of the viral assembly and life cycle, as well as the exploration of several viral functional proteins as potential therapeutic targets.

The life cycle of SARS-CoV-2 within the host cell starts with its genome translation into two polyproteins, pp1a and pp1ab, which are subsequently cleaved by two viral proteases, the main protease (Mpro) and the papain-like protease (PLpro), into 16 nonstructural proteins (NSP1–NSP16). NSP3 is the largest of these, with a molecular mass of approximately 200 kDa. As NSP3 is one of the few (poly)proteins which become functional at the start of the virus life cycle and as it is highly conserved in all coronaviruses (Lei *et al.*, 2018 ▸), NSP3 has been spotlighted as a key viral protein (Dable-Tupas & Derecho, 2022 ▸; Chan *et al.*, 2024 ▸). The intricate complexity of NSP3, which consists of approximately 15 different domains, gives the protein its versatile functions, including evasion of the host immune system and involvement in different steps of the viral replication cycle such as polyprotein maturation, double-membrane vesicle (DMV) formation and pore creation in the DMVs (Lei *et al.*, 2018 ▸; Wolff, Limpens *et al.*, 2020 ▸; Wolff, Melia *et al.*, 2020 ▸; Huang *et al.*, 2024 ▸). However, this complexity also poses challenges in obtaining high-resolution structural information for the development of therapeutics. Because the protease activity of the PLpro domain of NSP3 is of central importance for many of its critical functions above and because the PLpro domain is highly amenable for structural and fragment-binding studies compared with full-length NSP3, the PLpro domain has emerged as a potential therapeutic target.

PLpro and the adjacent ubiquitin-like domain 2 (Ubl2) together are often considered as a single protein (von Soosten *et al.*, 2022 ▸). The Ubl2–PLpro protomer (referred to as PLpro in this paper) is a 35 kDa papain-like cysteine protease that includes Ubl2 at its N-terminus followed by its catalytic core (Supplementary Fig. S1), which itself consists of three sub­domains (Osipiuk *et al.*, 2021 ▸): an α-helical ‘thumb’, a β-stranded ‘finger’ and a β-stranded ‘palm’. The fingers subdomain contains a zinc-binding site coordinated by four conserved cysteine residues (Cys189, Cys192, Cys224 and Cys226; Gao *et al.*, 2021 ▸) distributed within the loops between the β-strands of the finger domain (Supplementary Fig. S1). Mutational studies of SARS-CoV-1 have shown that the zinc-finger loop residues are essential for proper folding and solubility of the enzyme and for maintaining the integrity of the active site (Barretto *et al.*, 2005 ▸). The active centre is located in the palm domain and comprises a catalytic triad formed by the amino acids Cys111, His272 and Asp286 at the interface between the thumb and palm subdomains (Gao *et al.*, 2021 ▸). Asp286 promotes the deprotonation of Cys111 by His272, rendering it a more potent nucleophile (Osipiuk *et al.*, 2021 ▸). Cys111 then binds the substrate via a covalent thioether linkage, followed by substrate (peptide) cleavage (Rut *et al.*, 2020 ▸). PLpro recognizes the target tetrapeptide motif L*X*GG↓*XX* by blocking loop 2, BL2, situated in the palm domain between β-strands 11 and 12 (residues 267–271; Gao *et al.*, 2021 ▸).

The enzymatic protease activity of PLpro is responsible for the cleavage of the viral polyproteins pp1a and pp1ab, leading to the release of mature NSP1–NSP3 (von Soosten *et al.*, 2022 ▸). The rest of the polyprotein cleavage to produce NSP4–NSP15 is performed by the main protease (MPro; Gao & Zhang, 2025 ▸). PLpro harbours two distinct substrate-binding sites, SUb1 and SUb2 (Supplementary Fig. S1), each with unique specificity and activity (Bosken *et al.*, 2020 ▸). Notably, the SUb1 site primarily interacts with ubiquitin, ubiquitin-like proteins and ISG15, while the SUb2 site accommodates polyubiquitin-linked chains (von Soosten *et al.*, 2022 ▸). The binding capability of PLpro to these proteins underscores its direct involvement in evading the host immune response, a mechanism shared with other coronaviruses such as Middle East respiratory syndrome-related coronavirus (MERS-CoV) (Bailey-Elkin *et al.*, 2014 ▸; Clasman *et al.*, 2020 ▸). Consequently, these two sites emerge as promising targets for drug discovery.

In spite of the importance and technical accessibility of PLpro for structural therapeutic studies, no clinically approved drugs have been developed against it to date (Chan *et al.*, 2024 ▸). One important factor to consider is the accessibility of its drug-binding sites *in situ*. Although most crystal structures of PLpro deposited in the Protein Data Bank show the Ubl2 domain in a single, linearly aligned conformation, molecular-dynamics simulations indicate considerable intrinsic mobility of Ubl2 and the adjacent loops, including the BL2 and zinc-binding loops (Bosken *et al.*, 2020 ▸; Báez-Santos *et al.*, 2015 ▸). Consistent with this, recent *in situ* cryo-electron tomography of the NSP3–NSP4 pore reveals a markedly different (∼45° bent) Ubl2 orientation (Huang *et al.*, 2024 ▸). Although this orientation is distinct from the linear arrangement seen in most isolated PLpro crystal structures, the overall picture emerging from structural and computational studies points to pronounced conformational plasticity of this domain. In our 1.95 Å resolution crystal structure of SARS-CoV-2 PLpro, two protomers in the asymmetric unit adopt different Ubl2 orientations, namely ‘closed’ and ‘open’. The ‘closed’ conformation corresponds to the canonical orientation described in most SARS-CoV PLpro structures, whereas the ‘open’ conformation is rotated and features an ∼4 Å displacement with respect to the closed form. Here, we present a detailed analysis of this previously uncharacterized conformation of PLpro from SARS-CoV-2 in comparison to the closed state and provide an overview of all crystal structures reported so far with respect to Ubl2 conformational variability. The diversity of Ubl2 positioning observed across crystallographic and *in situ* structures underscores the intrinsic mobility of the domain and suggests that PLpro can access multiple conformational states depending on its molecular context.

## 2. Materials and methods

### 2.1. PLpro recombinant protein production

The SARS-CoV-2 PLpro wild-type (WT) and C111S mutant *Escherichia coli* codon-optimized genes inserted into a pET-28a(+) vector were synthesized by GenScript (https://www.genscript.com). The bacterial vector contains a C-terminal TEV protease cleavage site and a His_6_ polyhistidine tag (Supplementary Fig. S1). Plasmids were amplified using the *E. coli* MACH1 strain and were verified by DNA sequencing at Eurofins (https://www.eurofinsus.com/genomic-services/dna-sequencing). The final protein sequences are shown in Table 1 ▸.

Protein expression was performed in *E. coli* BL21(DE3) cells. Cell cultures were grown at 37°C until the optical density at 600 nm (OD_600_) reached 0.6. Expression was induced by adding isopropyl β-d-1-thiogalactopyranoside to a final concentration of 1 m*M* and ZnSO_4_ to 0.1 m*M*, followed by a temperature decrease to 18°C for ∼20 h. The cells were harvested via centrifugation at 5000*g* at 4°C. The cell pellets were resuspended in lysis buffer [50 m*M* HEPES pH 7.5, 500 m*M* NaCl, 5 m*M* β-mercaptoethanol (β-ME), 10 µ*M* ZnCl_2_, 5% glycerol, 0.5% Tween, lysozyme and a complete protease-inhibitor EDTA-free tablet (Roche)] and lysed by sonication on ice. The lysate was centrifuged for 20 min at 40 000*g* at 4°C. The supernatant was loaded onto a 5 ml HisTrap column (GE Healthcare) for affinity-chromatography purification at 4°C. The target protein was eluted from the column with elution buffer (50 m*M* HEPES pH 7.5, 500 m*M* NaCl, 5 m*M* β-ME, 10 µ*M* ZnCl_2_, 500 m*M* imidazole). TEV protease was added in a 1:20(*w*/*w*) ratio to the protein for His-tag cleavage and dialysed overnight at 4°C into 50 m*M* HEPES pH 7.5, 300 m*M* NaCl, 5 m*M* β-ME, 10 µ*M* ZnCl_2_. A second HisTrap run was performed and the flowthrough containing the His-tag-cleaved protein was collected and dialysed again overnight at 4°C with 50 m*M* HEPES pH 7.5, 150 m*M* NaCl, 5 m*M* β-ME, 10 µ*M* ZnCl_2_. The protein was then further purified by size-exclusion chromatography using a Superdex 75 10/300 GL column (Cytiva) at 4°C with final buffer consisting of 50 m*M* HEPES pH 7.5, 150 m*M* NaCl, 1 m*M* TCEP, 10 µ*M* ZnCl_2_. Protein-containing elution peaks were pooled and concentrated using a centrifugal filter unit (Amicon Ultra-4, 3.5 kDa molecular-weight cutoff) to around 10 mg ml^−1^. Concentrated proteins were flash-frozen using liquid nitrogen and stored at −80°C, ready to be used for crystallization or other downstream experiments.

### 2.2. Crystallization

The purified protein was used in crystallization trials at a concentration of 10 mg ml^−1^. Initial intergrown crystals were obtained for the PLpro^C111S^ mutant using Grid Screen Salt (Hampton Research) in a condition consisting of 0.1 *M* bicine pH 9, 1.6 *M* ammonium sulfate in sitting drops set up by the EMBL Grenoble HTX crystallization facility (Dimasi *et al.*, 2007 ▸; Márquez & Cipriani, 2014 ▸). Single crystals of the PLpro^C111S^ mutant were obtained by refining the starting condition to 0.1 *M* bicine pH 8, 1.45 *M* ammonium sulfate, 10% glycerol in 24-well plates and performing micro-seeding (Table 2 ▸). Seeds were made from crystals of the PLpro^C111S^ mutant using the Hampton Research seed bead kit following the manufacturer’s protocol. Seed stock was prepared by crushing crystals in one crystallization drop and transferring this solution to a reaction tube with one seed bead and 50 µl reservoir solution. The tube was vortexed for 3 min, stopping every 30 s to cool the solution on ice for 20 s. New drops were set up with a dilution series of the seed stock ranging from a 50-fold to 1500-fold dilution in reservoir buffer. PLpro^WT^ crystals were obtained by cross-seeding with previously obtained PLpro^C111S^ crystals in a hanging-drop vapour-diffusion setup. The reservoir volume was 500 µl with a drop size of 2.5 µl (1 µl protein solution, 1 µl reservoir solution and 0.5 µl seed solution).

### 2.3. X-ray diffraction data collection and processing

Crystals originally grown without any glycerol in the crystallization buffer were screened for a suitable cryoprotectant. However, all cryoprotectants tested reduced the diffraction quality and the diffraction limit of the crystals. This was ultimately overcome by crystallizing the protein in the presence of 10% glycerol in the crystallization buffer, followed by directly flash-cooling the crystals in liquid nitrogen.

Diffraction data from three different crystal types were collected: PLpro^WT^ and PLpro^C111S^, both of which were harvested two months after the drops were set up, and PLpro^WT_‘aged’^, which was harvested eight months after crystallization setup. X-ray diffraction data were collected on beamline ID30B (McCarthy *et al.*, 2018 ▸) at the European Synchrotron Radiation Facility (ESRF), Grenoble, France, using an EIGER2 X 9M detector at 100 K at a wavelength of 0.873 Å for PLpro^WT^ and PLpro^C111S^ and 1.282 Å for PLpro^WT_‘aged’^ (Table 3 ▸). Data processing was performed using *autoPROC* and *STARANISO* (Vonrhein *et al.*, 2011 ▸, 2024 ▸).

### 2.4. Structure solution and refinement

The structure-solution and refinement statistics are listed in Table 4 ▸. All structures were solved by molecular replacement using *Phenix* (Liebschner *et al.*, 2019 ▸). The search model for the C111S mutant structure was the PLpro structure from SARS-CoV-2 (PDB entry 7d7k; Zhao *et al.*, 2021 ▸). The WT structures were solved using the C111S mutant structure reported here as a search model. Model refinement was performed using *Coot* and *phenix.refine* (Emsley *et al.*, 2010 ▸; Liebschner *et al.*, 2019 ▸). Experimental phases using the anomalous scattering contribution of zinc at an energy of 9.67 keV of the PLpro^WT_’aged’^ crystal were calculated with *SFALL*, merged with the MTZ file using *CAD* and the anomalous difference Fourier map was subsequently generated with fast Fourier transform (all as part of the *CCP*4 suite; Agarwal, 1978 ▸; Immirzi, 1966 ▸; Potterton *et al.*, 2003 ▸; Agirre *et al.*, 2023 ▸; Ten Eyck, 1973 ▸; Read & Schierbeek, 1988 ▸).

### 2.5. SAXS data collection, processing and modelling

SEC-SAXS measurements were carried out on BM29 at the ESRF (Tully *et al.*, 2023 ▸); in brief, 100 µl of 10 mg ml^−1^ PLpro^C111S^ was loaded onto a Superdex 75 10/300 column (Cytiva) pre-equilibrated with five column volumes of running buffer (50 m*M* HEPES pH 7.5, 150 m*M* NaCl, 1 m*M* TCEP, 10 µ*M* ZnCl_2_). SAXS data were collected using X-rays of wavelength 0.9919 Å (12.5 keV) and a sample-to-detector distance of 2.81 m, corresponding to a *q*-range of 0.007–0.5 Å^−1^. 1400 frames were collected at 2 s per frame. Data were initially processed using the BM29 automated software pipeline *FreeSAS* (Kieffer *et al.*, 2022 ▸) with additional post-processing using *ScatterIV* (Tully *et al.*, 2021 ▸). Flexibility was measured using *BilboMD* (Pelikan *et al.*, 2009 ▸), in which molecular-dynamics (MD) simulations are used to explore conformational space and provide an ensemble of molecular models from which a SAXS curve is calculated and compared with the experimental curve. Residues 57–60 of PLpro^C111S^ were allowed to be flexible between two rigid bodies.

## 3. Results and discussion

### 3.1. Crystallization and X-ray diffraction of PLpro WT and the active-site mutant C111S

**Figure 1:**
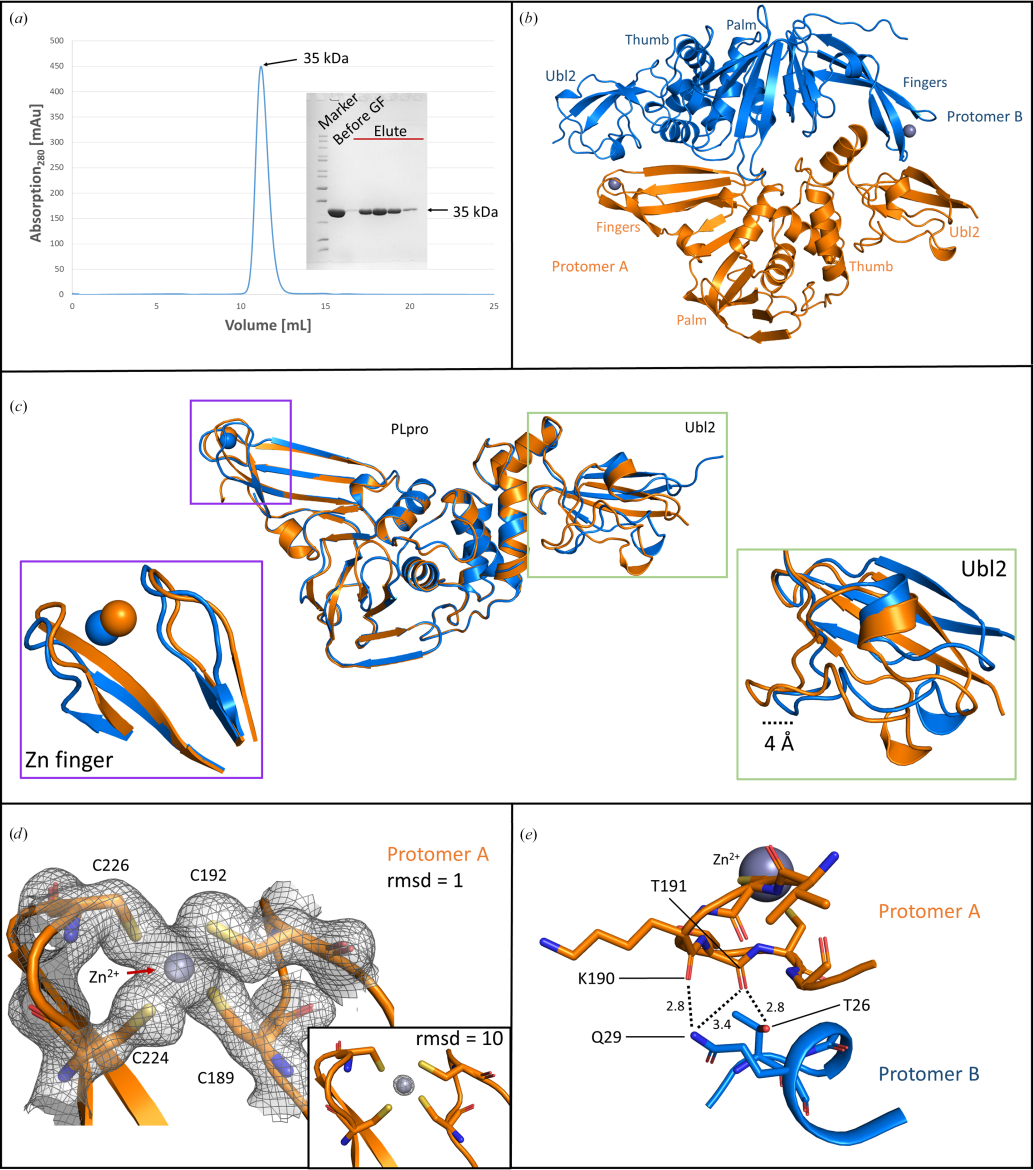
(*a*) SEC elution purification profile of PLpro^WT^ showing a single peak corresponding to the monomeric size and SDS–PAGE confirming the purity of the target protein. (*b*) Ribbon-diagram representation of the asymmetric unit containing two protomers. Protomer *A* is represented in orange and protomer *B* in blue. The thumb, finger, palm and Ubl2 domains are marked. (*c*) Cartoon-diagram representation of differences between protomer *A* and *B* [using the same colour scheme as shown in (*b*)] of the asymmetric unit, showing the differences in two different insets for the zinc-finger and the Ubl2 domain (from left to right). (*d*) Electron density of the protomer *A* zinc finger showing the coordination of the ion by the four cysteine residues and an inset showing the density around the zinc ion present at a contour level of 10 r.m.s.d.. (*e*) Interactions between the protomer *A* zinc-finger and protomer *B* Ubl2 domain. Amino acids involved in hydrogen bonds are labelled and the hydrogen bonds are marked as black dashed lines.

Both PLpro protein constructs, PLpro^WT^ and PLpro^C111S^, were expressed as a C-terminal His-tag fusion protein. The His-tag was subsequently cleaved by TEV protease prior to crystallization. During expression of the protein, 100 m*M* ZnSO_4_ was added to the cell culture and 10 m*M* ZnCl_2_ was added to each purification buffer, which led to an increase in protein yields. Both PLpro^WT^ and PLpro^C111S^ proteins eluted as monomers in size-exclusion chromatography (SEC; Fig. 1 ▸*a*). However, analytical ultracentrifugation (AUC) also showed a small population of <2% with a molecular weight of 70.2 ± 2.5 kDa, corresponding to a PLpro dimer (71.4 kDa; Supplementary Fig. S2). In spite of the similar behaviour of both constructs in SEC and AUC, only the C111S mutant crystallized readily. However, the initial crystals only diffracted to approximately 4 Å resolution. We were able to optimize the mutant crystals by micro-seeding using crystals obtained from an initial crystallization screen. Crystallization of the WT protein was achieved through cross-seeding, starting from a seed stock prepared from the optimized mutant crystals. While these crystals diffracted to below 3 Å resolution when directly flash-cooled in liquid nitrogen, they showed considerably degraded diffraction quality upon the addition of cryo­protectants. This limitation was overcome by incorporating 10% glycerol in the crystallization buffer, followed by direct flash-cooling of the obtained crystals in liquid nitrogen. Under these conditions, the crystals consistently diffracted to better than 2 Å resolution and the structures of PLpro^WT^ and PLpro^C111S^ were resolved in the orthorhombic space group *P*2_1_2_1_2 with very similar unit-cell dimensions (Table 3 ▸). Each structure contains two molecules in the asymmetric unit, with Matthews coefficients of 2.66 and 2.58 Å^3^ Da^−1^ for PLpro^WT^ and PLpro^C111S^, respectively, indicating solvent contents of 54% and 52% in the crystals.

Unless specified, all descriptions given here are for the PLpro^WT^ crystals. The two protomers in the asymmetric unit are organized in an almost dimeric arrangement (Fig. 1 ▸*b*). For protomer *A*, residues 3–322 were modelled into the electron density, while protomer *B* showed clearly interpretable electron density for residues 1–315 of the total 322 residues. Globally, the two protomers show almost identical structures (r.m.s.d. of 0.36 Å for all C^α^ atoms in common). However, local differences are visible, especially in the two functionally important regions known for their dynamic nature: the Ubl2 domain and the zinc-binding loop (Frieman *et al.*, 2009 ▸; Bosken *et al.*, 2020 ▸; Báez-Santos *et al.*, 2015 ▸).

### 3.2. Open and closed conformations of the Ubl2 domain

**Figure 2:**
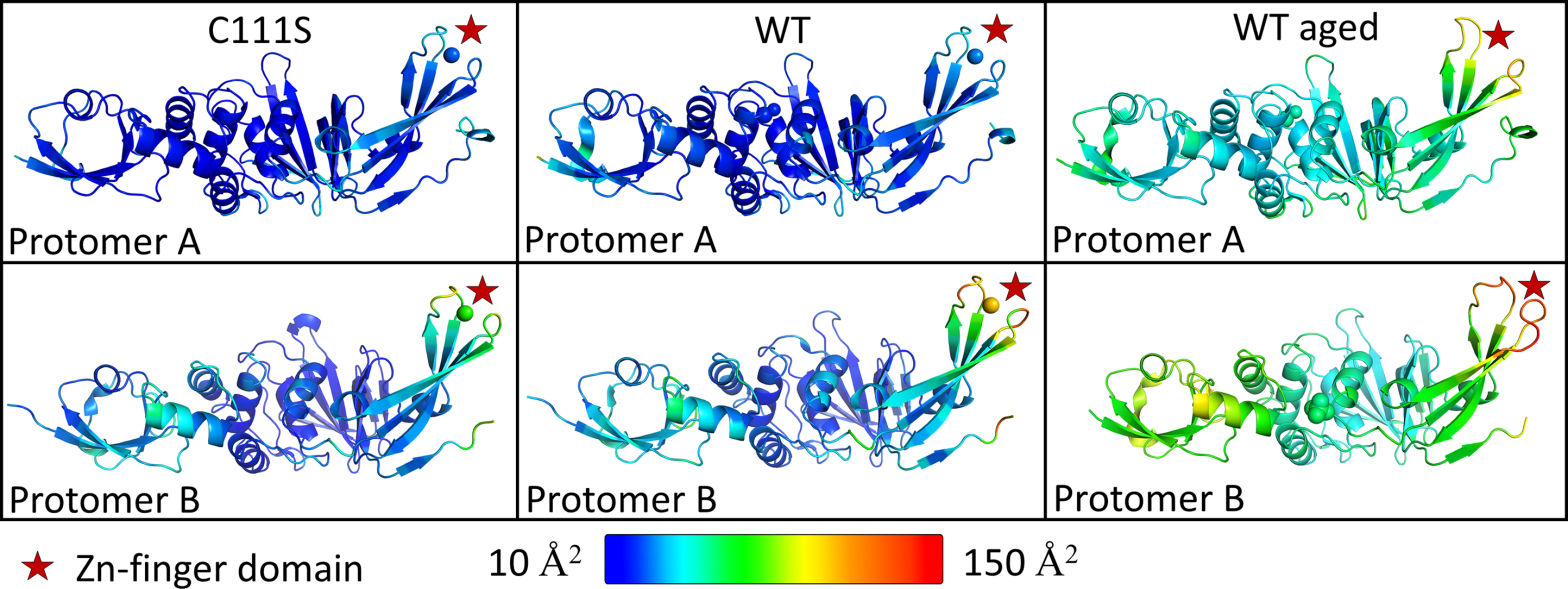
Representation of *B* factors in a rainbow colour spectrum (ranging from 10 to 150 Å^2^) of all three structures for protomer *A* and protomer *B*. Higher *B* factors can be observed for protomer *B* zinc-finger regions, especially for the PLpro^WT_‘aged’^ structure that has no zinc bound.

The Ubl2 domain is a ubiquitous domain associated with all coronavirus PLpro proteins and has been shown to be essential for the stability and catalytic efficiency of PLpro in SARS-CoV-1, SARS-CoV-2 and murine hepatitis virus (Frieman *et al.*, 2009 ▸; Mielech *et al.*, 2015 ▸; Arya *et al.*, 2025 ▸). It is a small domain comprising approximately 60 amino acids attached to the ridge helix (residues 61–68) of the thumb domain of PLpro. In both the PLpro^WT^ and PLpro^C111S^ structures reported here, we observe distinct conformations of the Ubl2 domain in the two protomers. The Ubl2 domain of protomer *B* has undergone a rotation and is in a different position relative to the ‘ridge’ helix (Asp62–His73) compared with protomer *A* (Fig. 1 ▸*c*, Ubl2 inset). This rotation places the Ubl2 domain of chain *B* spatially further away from the PLpro domain, defining what we refer as the ‘open’ conformation. A maximum displacement of 3.9 Å is observed between the two protomers at around residue Ala39 (C^α^), highlighting a notable structural rearrangement that distinguishes protomer *B* from protomer *A*. This positioning of the Ubl2 domain in protomer *B* cannot be attributed to crystal packing as the Ubl2 domain does not engage in lattice interactions, except for the N-terminal tip, which contacts a short loop (residues 293–297) of a symmetry-related molecule (Supplementary Fig. S3). Moreover, the Ubl2 residues of this monomer show higher *B*-factor values than those of protomer *A* (Fig. 2 ▸ and Supplementary Fig. S4). To investigate flexibility in the solution state, SAXS measurements were taken. The SAXS results from PLpro^C111S^ show a monomeric globular protein with a radius of gyration (*R*_g_) of 24.98 Å and a maximum dimension (*D*_max_) of 98 Å (Supplementary Fig. S5). To explore any flexibility, the *BilboMD* software (Pelikan *et al.*, 2009 ▸) was used to create an ensemble of molecular models that conformationally sample the molecular space. An initial fit to the monomeric PLpro^C111S^ structure was taken (χ^2^ = 2.33), the linker region between the Ubl2 domain was then allowed freedom to move and the resulting ensemble of models generated indicate that the Ubl2 domain does have movement about the linker region (Supplementary Fig. S6); a three-state model gave the best fit (χ^2^ = 1.40). Thus, this supports the proposal that localized flexibility may arise from the Ubl2 domain about a relatively rigid PLPro core domain. Importantly, the SAXS data are well described by monomeric models, supporting that the observed flexibility in the Ubl2 domain reflects intrinsic conformational variability rather than intermolecular interactions as in the crystal.

The conformation observed in protomer *A*, which we refer to as ‘closed’, has been commonly observed, with slight variations, in several SARS-CoV-1 and SARS-CoV-2 PLpro structures deposited in the Protein Data Bank (PDB entry 2fe8, Ratia *et al.*, 2006 ▸; PDB entry 7nfv, Srinivasan *et al.*, 2022 ▸). Both Ubl2 conformations seem to exhibit hydrogen-bond interactions with PLpro. In protomer *A* (the closed state), hydrogen bonds are observed between His17 and Glu67 (3.1 Å) and between Asp37 and Lys91 (2.8 Å). In contrast, the open conformation in protomer *B* maintains the hydrogen bond between His17 and Glu67 (2.8 Å) but lacks the interaction between Asp37 and Lys91. Instead, a new hydrogen bond forms between Gly38 and Lys91 (3.3 Å). In SARS-CoV-1 (for example PDB entry 5y3e), equivalent hydrogen bonds occur between His17 and Glu67 (2.8 Å) and Asp37 and Lys91 (2.8 Å), as observed in protomer *A* (Lin *et al.*, 2018 ▸). The presence of only two hydrogen bonds between the Ubl2 and catalytic domains of PLpro may be considered weaker but is perhaps necessary to accommodate the conformational flexibility of the Ubl2 domain (Bosken *et al.*, 2020 ▸; Báez-Santos *et al.*, 2015 ▸). Nevertheless, in both conformations residues involved in SUb2, which is located proximal to the Ubl2 domain, appear to be structurally conserved, suggesting that the open conformation is unlikely to impact substrate binding at this site.

The ‘closed’ conformation is prevalent across all known SARS-CoV-2 PLpro apo structures (13 in total; PDB entries 6w9c, 6wrh, 6wzu, 6xg3, 7d47, 7d6h, 7d7k, 7cjd, 7nfv, 7ybg, 8fwn, 8fwo and 8vec), whether crystallized as monomers or as dimers. However, to our knowledge, the ‘open’ conformation observed in our structure has not been reported in any of these structures. Interestingly, one SARS-CoV-2 PLpro structure (PDB entry 7d7k; Zhao *et al.*, 2021 ▸), crystallized in space group *P*6_5_22 with two molecules in the asymmetric unit, reveals one protomer in the ‘closed’ conformation and the other in an intermediate state positioned between the ‘open’ conformation observed in our protomer *B* and the ‘closed’ conformation (Supplementary Fig. S7). The observation of these conformations across different space groups and different protomer–protomer packings reduces the likelihood that the observation is linked to crystal-packing artefacts and confirms the existing notion of flexibility within the Ubl2 domain (Bosken *et al.*, 2020 ▸).

### 3.3. Flexibility of the zinc-binding loop

Next, we analysed the flexibility of the zinc-binding loop and its correlation to zinc occupancy in both protomers. Interestingly, protomer *A* of PLpro^WT^ has a fully occupied zinc ion coordinated by four conserved cysteine residues, Cys189, Cys192, Cys224 and Cys226 (Fig. 1 ▸*d* and inset). The electron density for the zinc ion is clearly visible at a contour level exceeding 12 r.m.s.d., with a corresponding *B* factor of 28 Å^2^. The surrounding loop residues in protomer *A* exhibit defined electron density and low *B* factors (for example, the *B* factor of Cys189 is 29 Å^2^; Table 5 ▸). In contrast, protomer *B* shows only weak electron density for the zinc ion (disappearing at contour levels of >3 r.m.s.d.) with a significantly higher *B* factor of 110 Å^2^. The associated zinc-binding loop residues in protomer *B* also display elevated *B* factors (see Table 5 ▸). A similar situation is observed in the PLpro^C111S^ structure, where the *B* factors of these residues in protomers *A* and *B* remain comparable to those of PLpro^WT^. In our hands, the removal of bound zinc by EDTA in the purification buffer resulted in protein precipitation, while addition of zinc to the cell culture and the purification buffer increased the protein yield (data not shown). These observations support the notion that zinc binding to PLpro is essential for stabilizing the flexibility of the zinc-binding loop and thereby maintaining the structural integrity of the protein (Jiang *et al.*, 2022 ▸).

We extended this analysis to explore whether zinc-ion occupancy and zinc-binding loop stability contribute to the conformational variability observed in the Ubl2 domain in the asymmetric unit. In our structures, both protomers are arranged such that the N-terminal region of one protomer is positioned near the C-terminal region of the other, although not symmetrically, forming a configuration reminiscent of a ‘yin–yang’ pattern. This arrangement positions the zinc-binding loop of protomer *A* to interact with the Ubl2 domain of protomer *B* (Fig. 1 ▸*e*). Specifically, there are hydrogen bonds from the main-chain O atom of Thr191 (in the zinc-binding loop) in protomer *A* to the OG1 atom of Thr26 and the NE2 atom of Gln29 in protomer *B*. Additionally, the main-chain O atom of Lys190 (in the zinc-binding loop) in protomer *A* forms a hydrogen bond to the NE2 atom of Gln29 in protomer *B*. In contrast, the zinc-binding loop of protomer *B* does not form any interactions with the Ubl2 domain of protomer *A*. This suggests that the interaction with a stable zinc-binding loop could lock the Ubl2 domain in this open conformation, however in a specific environment such as our crystal packing. This raised the question: would removal of the zinc ion from protomer *A* weaken its interaction with the Ubl2 domain of protomer *B*, thereby inducing it to adopt a ‘closed’ conformation? To answer this question, we examined PLpro^WT^ crystals that were 7–8 months old, based on the fact that zinc binding to proteins is pH-dependent (Kluska *et al.*, 2018 ▸). Over time, we noticed that the pH of our crystallization drops shifted from basic to acidic (as measured with pH paper). To assess the presence of zinc within these ‘aged’ crystals, we performed X-ray anomalous scattering experiments slightly above the zinc absorption edge (9.7 keV), which showed no measurable anomalous signal at the zinc-binding site. Diffraction data collected from several PLpro^WT_’aged’^ crystals showed weak electron density at the zinc-binding site and ill-defined electron density for zinc-binding-loop residues, with elevated *B* factors for both protomers (Fig. 2 ▸ and Supplementary Figs. S4, S8 and S9). As anticipated, the Ubl2 domain of protomer *B* was no longer in an ‘open’ conformation in this structure, but adopted an ‘intermediate’ conformation between the open and the closed states (Supplementary Fig. S8). Interestingly, the crystals remained intact despite a minor reduction in the resolution obtained, implying that they can tolerate the conformational variability of Ubl2.

While this is the case within the asymmetric unit arrangement, a detailed analysis of *B* factors within each protomer reveals a trend; elevated *B* factors of the zinc-binding loop residues correlate with higher *B* factors in the Ubl2 domain of the same monomer. This is also confirmed across several existing structures (Supplementary Figs. S4 and S10). The potential allosteric link between zinc-binding loop flexibility and Ubl2 conformational variability, along with the possibility that zinc ion binding regulates this variability, warrant further investigation.

### 3.4. Intrinsic conformational variability of Ubl2 in PLpro

To decipher whether the open form could be one of the physiologically relevant states of PLpro, we expanded our search to PLpro structures from other coronaviruses. Our analyses showed that several MERS-CoV PLpro structures (PDB entries 5v69, 4rna, 4pt5, 4p16, 4rez and 4r3d) have a conformation similar to our ‘open’ conformation (Supplementary Figs. S1 and S11), highlighting the possibility that the ‘open’ conformation represents an intrinsic state of PLpro and may be relevant to consider in future inhibitor studies. This is the first time that this conformation has been characterized from crystallographic analyses for SARS-CoV-1 and SARS-CoV-2. Further supporting the physiological relevance of Ubl2 flexibility, a recent cryo-EM structure of the NSP3–NSP4 pore complex manifested an altogether different conformation for Ubl2, differing from the reported open or closed states, which exhibited unanticipated interactions with C-terminal domains of NSP3 (Y1-CoVG; Huang *et al.*, 2024 ▸). While the crystal and cryo-EM structures provide valuable snapshots of Ubl2 conformational variability, they do not capture its full dynamic behaviour. Additional approaches such as molecular-dynamics simulations could further explore the conformational landscape of the Ubl2 domain, particularly in relation to ligand and substrate binding and its potential allosteric coupling to the zinc-binding site.

Together, these findings show the essential nature of Ubl2 flexibility in the structural and functional integrity of the papain-like protease of SARS-CoV-2. We believe that this intrinsic flexibility of the Ubl2 domain of PLpro, which may open up a pocket for potential inhibitors, could be exploited in hit- and lead-finding campaigns to identify starting points for antiviral drug development.

## 4. Related literature

The following reference is cited in the supporting information for this article: Robert *et al.* (2025 ▸).

## Supplementary Material

PDB reference: SARS-CoV2 papain-like protease, wild type, 9hhg

PDB reference: zinc-depleted (aged crystals), 9hhi

PDB reference: C111S mutant, 9hhh

Supplementary Figures. DOI: 10.1107/S2053230X26003699/nq5003sup1.pdf

## Figures and Tables

**Table 1:** Amino-acid sequences of PLpro^WT^ and PLpro^C111S^ constructs In the amino-acid sequences, His-tag sites and TEV protease cleavage sites are indicated by underlined and bold characters, respectively. Residues in italics are derived from the cloning vector.

Complete amino-acid sequence of the PLpro^WT^ construct produced	*MG*EVRTIKVFTTVDNINLHTQVVDMSMTYGQQFGPTYLDGADVTKIKPHNSHEGKTFYVLPNDDTLRVEAFEYYHTTDPSFLGRYMSALNHTKKWKYPQVNGLTSIKWADNNCYLATALLTLQQIELKFNPPALQDAYYRARAGEAANFCALILAYCNKTVGELGDVRETMSYLFQHANLDSCKRVLNVVCKTCGQQQTTLKGVEAVMYMGTLSYEQFKKGVQIPCTCGKQATKYLVQQESPFVMMSAPPAQYELKHGTFTCASEYTGNYQCGHYKHITSKETLYCIDGALLTKSSEYKGPITDVFYKENSYTTTIKP**ENLYFQG***LE*HHHHHH
Complete amino-acid sequence of the PLpro^C111S^ construct produced	*MG*EVRTIKVFTTVDNINLHTQVVDMSMTYGQQFGPTYLDGADVTKIKPHNSHEGKTFYVLPNDDTLRVEAFEYYHTTDPSFLGRYMSALNHTKKWKYPQVNGLTSIKWADNNSYLATALLTLQQIELKFNPPALQDAYYRARAGEAANFCALILAYCNKTVGELGDVRETMSYLFQHANLDSCKRVLNVVCKTCGQQQTTLKGVEAVMYMGTLSYEQFKKGVQIPCTCGKQATKYLVQQESPFVMMSAPPAQYELKHGTFTCASEYTGNYQCGHYKHITSKETLYCIDGALLTKSSEYKGPITDVFYKENSYTTTIKP**ENLYFQG***LE*HHHHHH

**Table 2:** Crystallization of PLpro^WT^ and PLpro^C111S^ constructs

Method	Hanging-drop vapour diffusion
Temperature (K)	291
Protein concentration (mg ml^−1^)	10
Buffer composition of protein solution	50 m*M* HEPES pH 7.5, 150 m*M* NaCl, 1 m*M* TCEP, 10 µ*M* ZnCl_2_
Composition of reservoir solution	1.45 *M* (NH_4_)_2_SO_4_, 0.1 *M* bicine pH 8, 10% glycerol
Volume and ratio of drop (C111S)	2.0 µl drop (1:1 protein:reservoir)
Volume and ratio of drop (WT)	2.5 µl drop (2:2:1 protein:reservoir:seed)

**Table 3:** X-ray data-collection and processing statistics Values in parentheses are for the outer shell.

	PLpro^C111S^	PLpro^WT^	PLpro^WT_‘aged’^
PDB code	9hhh	9hhg	9hhi
Wavelength (Å)	0.873	0.873	1.282
Rotation angle per image (°)	0.2	0.1	0.2
Total rotation range (°)	180	180	360
Exposure time per image (s)	0.02	0.05	0.01
Space group	*P*2_1_2_1_2	*P*2_1_2_1_2	*P*2_1_2_1_2
*a*, *b*, *c* (Å)	98.4, 106.3, 72.0	100.1, 106.5, 72.3	99.7, 106.7, 71.8
Solvent content (%)	52.4	53.8	53.1
Mosaicity (°)	0.15	0.2	0.2
Resolution range (Å)	72.21–1.63 (1.84–1.63)	72.90–1.82 (1.94–1.82)	72.87–2.29 (2.59–2.29)
Total No. of reflections	423584 (22142)	408638 (21944)	326646 (15180)
No. of unique reflections	62300 (3115)	59988 (2999)	24306 (1215)
Completeness			
Spherical (%)	65.5 (10.9)	85.4 (24.9)	69.4 (11.6)
Ellipsoidal (%)	95.4 (70.5)	94.9 (57.1)	93.9 (56.3)
Multiplicity	6.8 (7.1)	6.8 (7.3)	13.4 (12.5)
〈*I*/σ(*I*)〉	6.7 (1.7)	6.6 (1.3)	9.6 (1.5)
CC_1/2_	0.996 (0.595)	0.967 (0.810)	0.997 (0.591)
*R*_meas_ (all *I*+ and *I*−)	0.162 (1.687)	0.171 (2.057)	0.232 (1.930)
Overall *B* factor from Wilson plot (Å^2^)	18.1	25.4	41.6

**Table 4:** Structure-solution and refinement statistics The resolution of the datasets has been lowered in refinement compared with the initially processed datasets in order to achieve a completeness of >80% for the high-resolution shell. Values in parentheses are for the outer shell.

	PLpro^C111S^	PLpro^WT^	PLpro^WT_’aged’^
Resolution range (Å)	72.21–1.98 (2.02–1.98)	72.94–1.95 (1.98–1.95)	72.87–2.70 (2.84–2.70)
Completeness (%)	98.48 (80.93)	98.08 (82.35)	98.57 (89.94)
No. of reflections, working set	52496 (2359)	55966 (2291)	21404 (2737)
No. of reflections, test set	2458 (122)	2756 (127)	1024 (140)
Final *R*_cryst_	0.179 (0.231)	0.187 (0.2538)	0.224 (0.3119)
Final *R*_free_	0.215 (0.267)	0.221 (0.3453)	0.284 (0.3459)
No. of non-H atoms			
Total	5599	5667	5208
Protein	5099	5070	5016
Ligand	40	16	18
Water	460	581	174
Total	5599	5667	5208
R.m.s. deviations			
Bond lengths (Å)	0.009	0.007	0.002
Angles (°)	0.90	0.82	0.43
Average *B* factors (Å^2^)			
Overall	26.01	30.8	59.3
Protein	25.4	30.3	59.6
Ligand	43.0	48.8	49.4
Water	31.0	34.7	52.1
Ramachandran plot			
Most favoured (%)	97.0	96.7	96.2
Allowed (%)	3.0	3.3	3.8

**Table 5:** *B* factors (Å^2^) of zinc ions in the zinc-binding loop, the cysteine residues in the loop and the overall structure for different structures deposited in the PDB as well as for the three structures presented in this article (PLpro^WT^, PLpro^C111S^ and PLpro^WT_‘aged’^)

	Zn	Cys189	Cys192	Cys224	Cys226	Overall	Resolution (Å)
PDB entry 7nfv	35	33	42	36	36	34	1.42
PDB entry 6wzu	41	41	50	43	43	39	1.79
PDB entry 7ybg	43	47	50	43	44	34	1.90
PDB entry 7d7k	45	47	65	68	75	47	1.90
PDB entry 7d47	94	82	76	93	80	40	1.97
PLpro^WT^, protomer *A*	28	29	32	31	34	31	1.95
PLpro^WT^, protomer *B*	111	88	96	100	110		1.95
PLpro^C111S^, protomer *A*	26	26	29	28	29	26	1.98
PLpro^C111S^, protomer *B*	77	74	76	87	112		1.98
PLpro^WT_‘aged’^, protomer *A*	—	97	125	83	111	59	2.70
PLpro^WT_‘aged’^, protomer *B*	—	123	124	115	126		2.70

## Data Availability

The atomic coordinates and structure-factor amplitudes of the reported structures have been deposited in the Protein Data Bank under accession codes 9hhg for PLpro^WT^, 9hhh for PLpro^C111S^ and 9hhi for PLpro^WT_’aged’^.

## References

[bb1] Agarwal, R. C. (1978). *Acta Cryst.* A**34**, 791–809.

[bb2] Agirre, J., Atanasova, M., Bagdonas, H., Ballard, C. B., Baslé, A., Beilsten-Edmands, J., Borges, R. J., Brown, D. G., Burgos-Mármol, J. J., Berrisford, J. M., Bond, P. S., Caballero, I., Catapano, L., Chojnowski, G., Cook, A. G., Cowtan, K. D., Croll, T. I., Debreczeni, J. É., Devenish, N. E., Dodson, E. J., Drevon, T. R., Emsley, P., Evans, G., Evans, P. R., Fando, M., Foadi, J., Fuentes-Montero, L., Garman, E. F., Gerstel, M., Gildea, R. J., Hatti, K., Hekkelman, M. L., Heuser, P., Hoh, S. W., Hough, M. A., Jenkins, H. T., Jiménez, E., Joosten, R. P., Keegan, R. M., Keep, N., Krissinel, E. B., Kolenko, P., Kovalevskiy, O., Lamzin, V. S., Lawson, D. M., Lebedev, A. A., Leslie, A. G. W., Lohkamp, B., Long, F., Malý, M., McCoy, A. J., McNicholas, S. J., Medina, A., Millán, C., Murray, J. W., Murshudov, G. N., Nicholls, R. A., Noble, M. E. M., Oeffner, R., Pannu, N. S., Parkhurst, J. M., Pearce, N., Pereira, J., Perrakis, A., Powell, H. R., Read, R. J., Rigden, D. J., Rochira, W., Sammito, M., Sánchez Rodríguez, F., Sheldrick, G. M., Shelley, K. L., Simkovic, F., Simpkin, A. J., Skubak, P., Sobolev, E., Steiner, R. A., Stevenson, K., Tews, I., Thomas, J. M. H., Thorn, A., Valls, J. T., Uski, V., Usón, I., Vagin, A., Velankar, S., Vollmar, M., Walden, H., Waterman, D., Wilson, K. S., Winn, M. D., Winter, G., Wojdyr, M. & Yamashita, K. (2023). *Acta Cryst.* D**79**, 449–461.

[bb3] Arya, R., Ganesh, J., Prashar, V. & Kumar, M. (2025). *Biol. Direct*, **20**, 102. 10.1186/s13062-025-00690-3 PMC12495856 41044763

[bb4] Báez-Santos, Y. M., St John, S. E. & Mesecar, A. D. (2015). *Antiviral Res.* **115**, 21–38. 10.1016/j.antiviral.2014.12.015 PMC5896749 25554382

[bb5] Bailey-Elkin, B. A., Knaap, R. C. M., Johnson, G. G., Dalebout, T. J., Ninaber, D. K., van Kasteren, P. B., Bredenbeek, P. J., Snijder, E. J., Kikkert, M. & Mark, B. L. (2014). *J. Biol. Chem.* **289**, 34667–34682. 10.1074/jbc.M114.609644 PMC4263872 25320088

[bb6] Barretto, N., Jukneliene, D., Ratia, K., Chen, Z., Mesecar, A. D. & Baker, S. C. (2005). *J. Virol.* **79**, 15189–15198. 10.1128/JVI.79.24.15189-15198.2005 PMC1316023 16306590

[bb7] Bosken, Y. K., Cholko, T., Lou, Y.-C., Wu, K.-P. & Chang, C. A. (2020). *Front. Mol. Biosci.* **7**, 174. 10.3389/fmolb.2020.00174 PMC7417481 32850963

[bb9] Chan, J. F.-W., Yuan, S., Chu, H., Sridhar, S. & Yuen, K.-Y. (2024). *Nat. Rev. Microbiol.* **22**, 391–407. 10.1038/s41579-024-01036-y 38622352

[bb10] Clasman, J. R., Everett, R. K., Srinivasan, K. & Mesecar, A. D. (2020). *Antiviral Res.* **174**, 104661. 10.1016/j.antiviral.2019.104661 PMC7114298 31765674

[bb12] Dable-Tupas, G. & Derecho, C. M. P. (2022). *Coronavirus Drug Discovery*, edited by C. Egbuna, pp. 181–203. Amsterdam: Elsevier.

[bb13] Dimasi, N., Flot, D., Dupeux, F. & Márquez, J. A. (2007). *Acta Cryst.* F**63**, 204–208. 10.1107/S1744309107004903 PMC2330191 17329815

[bb14] Du, S., Hu, X., Li, P., Xu, S., Kim, M., Liu, X. & Zhan, P. (2026). *Sig. Transduct. Target. Ther.* **11**, 69. 10.1038/s41392-025-02539-7 PMC12932771 41735249

[bb15] Emsley, P., Lohkamp, B., Scott, W. G. & Cowtan, K. (2010). *Acta Cryst.* D**66**, 486–501. 10.1107/S0907444910007493 PMC2852313 20383002

[bb16] Frieman, M., Ratia, K., Johnston, R. E., Mesecar, A. D. & Baric, R. S. (2009). *J. Virol.* **83**, 6689–6705. 10.1128/JVI.02220-08 PMC2698564 19369340

[bb17] Gao, X., Qin, B., Chen, P., Zhu, K., Hou, P., Wojdyla, J. A., Wang, M. & Cui, S. (2021). *Acta Pharm. Sin. B*, **11**, 237–245. 10.1016/j.apsb.2020.08.014 PMC7467110 32895623

[bb18] Gao, Y. & Zhang, J. (2025). *Acta Pharm. Sin. B*, **15**, 4497–4510. 10.1016/j.apsb.2025.07.001 PMC12491685 41049734

[bb19] Huang, Y., Wang, T., Zhong, L., Zhang, W., Zhang, Y., Yu, X., Yuan, S. & Ni, T. (2024). *Nature*, **633**, 224–231. 10.1038/s41586-024-07817-y PMC11374677 39143215

[bb20] Immirzi, A. (1966). *Crystallographic Computing Techniques*, edited by F. R. Ahmed, p. 399. Copenhagen: Munksgaard.

[bb21] Jiang, H., Yang, P. & Zhang, J. (2022). *Front. Chem.* **10**, 822785. 10.3389/fchem.2022.822785 PMC8905519 35281561

[bb22] Kieffer, J., Brennich, M., Florial, J.-B., Oscarsson, M., De Maria Antolinos, A., Tully, M. & Pernot, P. (2022). *J. Synchrotron Rad.* **29**, 1318–1328. 10.1107/S1600577522007238 PMC9455220 36073892

[bb23] Kluska, K., Adamczyk, J. & Krężel, A. (2018). *Coord. Chem. Rev.* **367**, 18–64.

[bb24] Lei, J., Kusov, Y. & Hilgenfeld, R. (2018). *Antiviral Res.* **149**, 58–74. 10.1016/j.antiviral.2017.11.001 PMC7113668 29128390

[bb25] Liebschner, D., Afonine, P. V., Baker, M. L., Bunkóczi, G., Chen, V. B., Croll, T. I., Hintze, B., Hung, L.-W., Jain, S., McCoy, A. J., Moriarty, N. W., Oeffner, R. D., Poon, B. K., Prisant, M. G., Read, R. J., Richardson, J. S., Richardson, D. C., Sammito, M. D., Sobolev, O. V., Stockwell, D. H., Terwilliger, T. C., Urzhumtsev, A. G., Videau, L. L., Williams, C. J. & Adams, P. D. (2019). *Acta Cryst.* D**75**, 861–877. 10.1107/S2059798319011471 PMC6778852 31588918

[bb26] Lin, M.-H., Moses, D. C., Hsieh, C.-H., Cheng, S.-C., Chen, Y.-H., Sun, C.-Y. & Chou, C.-Y. (2018). *Antiviral Res.* **150**, 155–163. 10.1016/j.antiviral.2017.12.015 PMC7113793 29289665

[bb27] Márquez, J. A. & Cipriani, F. (2014). *Structural Genomics: General Applications*, edited by Y. W. Chen, pp. 197–203. Totowa: Humana Press.

[bb28] McCarthy, A. A., Barrett, R., Beteva, A., Caserotto, H., Dobias, F., Felisaz, F., Giraud, T., Guijarro, M., Janocha, R., Khadrouche, A., Lentini, M., Leonard, G. A., Lopez Marrero, M., Malbet-Monaco, S., McSweeney, S., Nurizzo, D., Papp, G., Rossi, C., Sinoir, J., Sorez, C., Surr, J., Svensson, O., Zander, U., Cipriani, F., Theveneau, P. & Mueller-Dieckmann, C. (2018). *J. Synchrotron Rad.* **25**, 1249–1260. 10.1107/S1600577518007166 PMC6038607 29979188

[bb29] Mielech, A. M., Deng, X., Chen, Y., Kindler, E., Wheeler, D. L., Mesecar, A. D., Thiel, V., Perlman, S. & Baker, S. C. (2015). *J. Virol.* **89**, 4907–4917. 10.1128/JVI.00338-15 PMC4403493 25694594

[bb30] Oktavianawati, I., Santoso, M., Bakar, M. F. A., Kim, Y.-U. & Fatmawati, S. (2023). *Appl. Biol. Chem.* **66**, 89.

[bb31] Osipiuk, J., Azizi, S.-A., Dvorkin, S., Endres, M., Jedrzejczak, R., Jones, K. A., Kang, S., Kathayat, R. S., Kim, Y., Lisnyak, V. G., Maki, S. L., Nicolaescu, V., Taylor, C. A., Tesar, C., Zhang, Y.-A., Zhou, Z., Randall, G., Michalska, K., Snyder, S. A., Dickinson, B. C. & Joachimiak, A. (2021). *Nat. Commun.* **12**, 743. 10.1038/s41467-021-21060-3 PMC7854729 33531496

[bb32] Pelikan, M., Hura, G. L. & Hammel, M. (2009). *Gen. Physiol. Biophys.* **28**, 174–189. 10.4149/gpb_2009_02_174 PMC3773563 19592714

[bb33] Potterton, E., Briggs, P., Turkenburg, M. & Dodson, E. (2003). *Acta Cryst.* D**59**, 1131–1137. 10.1107/s0907444903008126 12832755

[bb34] Ratia, K., Saikatendu, K. S., Santarsiero, B. D., Barretto, N., Baker, S. C., Stevens, R. C. & Mesecar, A. D. (2006). *Proc. Natl Acad. Sci. USA*, **103**, 5717–5722. 10.1073/pnas.0510851103 PMC1458639 16581910

[bb35] Read, R. J. & Schierbeek, A. J. (1988). *J. Appl. Cryst.* **21**, 490–495.

[bb36] Robert, X., Guillon, C. & Gouet, P. (2025). *Nucleic Acids Res.* **53**, W277–W282. 10.1093/nar/gkaf326 PMC12230654 40276967

[bb37] Rut, W., Lv, Z., Zmudzinski, M., Patchett, S., Nayak, D., Snipas, S. J., El Oualid, F., Huang, T. T., Bekes, M., Drag, M. & Olsen, S. K. (2020). *Sci. Adv.* **6**, eabd4596. 10.1126/sciadv.abd4596 PMC7567588 33067239

[bb38] Srinivasan, V., Brognaro, H., Prabhu, P. R., de Souza, E. E., Günther, S., Reinke, P. Y. A., Lane, T. J., Ginn, H., Han, H., Ewert, W., Sprenger, J., Koua, F. H. M., Falke, S., Werner, N., Andaleeb, H., Ullah, N., Franca, B. A., Wang, M., Barra, A. L. C., Perbandt, M., Schwinzer, M., Schmidt, C., Brings, L., Lorenzen, K., Schubert, R., Machado, R. R. G., Candido, E. D., Oliveira, D. B. L., Durigon, E. L., Niebling, S., Garcia, A. S., Yefanov, O., Lieske, J., Gelisio, L., Domaracky, M., Middendorf, P., Groessler, M., Trost, F., Galchenkova, M., Mashhour, A. R., Saouane, S., Hakanpää, J., Wolf, M., Alai, M. G., Turk, D., Pearson, A. R., Chapman, H. N., Hinrichs, W., Wrenger, C., Meents, A. & Betzel, C. (2022). *Commun. Biol.* **5**, 805. 10.1038/s42003-022-03737-7 PMC9366811 35953531

[bb39] Ten Eyck, L. F. (1973). *Acta Cryst.* A**29**, 183–191.

[bb40] Tully, M. D., Kieffer, J., Brennich, M. E., Cohen Aberdam, R., Florial, J. B., Hutin, S., Oscarsson, M., Beteva, A., Popov, A., Moussaoui, D., Theveneau, P., Papp, G., Gigmes, J., Cipriani, F., McCarthy, A., Zubieta, C., Mueller-Dieckmann, C., Leonard, G. & Pernot, P. (2023). *J. Synchrotron Rad.* **30**, 258–266. 10.1107/S1600577522011286 PMC9814054 36601945

[bb41] Tully, M. D., Tarbouriech, N., Rambo, R. P. & Hutin, S. (2021). *J. Vis. Exp.*, e61578. 10.3791/61578 33586708

[bb42] Vonrhein, C., Flensburg, C., Keller, P., Fogh, R., Sharff, A., Tickle, I. J. & Bricogne, G. (2024). *Acta Cryst.* D**80**, 148–158. 10.1107/S2059798324001487 PMC10910543 38411552

[bb43] Vonrhein, C., Flensburg, C., Keller, P., Sharff, A., Smart, O., Paciorek, W., Womack, T. & Bricogne, G. (2011). *Acta Cryst.* D**67**, 293–302. 10.1107/S0907444911007773 PMC3069744 21460447

[bb44] von Soosten, L. C., Edich, M., Nolte, K., Kaub, J., Santoni, G. & Thorn, A. (2022). *Crystallogr. Rev.* **28**, 39–61.

[bb45] Wolff, G., Limpens, R. W. A. L., Zevenhoven-Dobbe, J. C., Laugks, U., Zheng, S., de Jong, A. W. M., Koning, R. I., Agard, D. A., Grünewald, K., Koster, A. J., Snijder, E. J. & Bárcena, M. (2020). *Science*, **369**, 1395–1398. 10.1126/science.abd3629 PMC7665310 32763915

[bb46] Wolff, G., Melia, C. E., Snijder, E. J. & Bárcena, M. (2020). *Trends Microbiol.* **28**, 1022–1033. 10.1016/j.tim.2020.05.009 PMC7289118 32536523

[bb50] World Health Organization (2026). *COVID-19 Deaths|WHO COVID-19 Dashboard*. Geneva: World Health Organization. https://data.who.int/dashboards/covid19/cases.

[bb47] Zhao, Y., Du, X., Duan, Y., Pan, X., Sun, Y., You, T., Han, L., Jin, Z., Shang, W., Yu, J., Guo, H., Liu, Q., Wu, Y., Peng, C., Wang, J., Zhu, C., Yang, X., Yang, K., Lei, Y., Guddat, L. W., Xu, W., Xiao, G., Sun, L., Zhang, L., Rao, Z. & Yang, H. (2021). *Protein Cell*, **12**, 877–888. 10.1007/s13238-021-00836-9 PMC8052528 33864621

