# Supplementary material for: Structural analysis of the flexibility of the Ubl2 domain within the papain-like protease of SARS-CoV-2
Source: Acta Crystallogr F Struct Biol Commun. 2026 May 18;82(Pt 6):222–30. doi: 10.1107/S2053230X26003699 (PMC13224804; doi:10.1107/S2053230X26003699)
Supplement: Supplementary file 1 [file f-82-00222-sup1.pdf]

Acta Cryst F

STRUCTURAL BIOLOGY
COMMUNICATIONS

Volume 82 (2026)

Supporting information for article:

Structural analysis of the flexibility of the Ubl2 domain within the papain-like protease of SARS-CoV-2

Gian Luca Freiherr von Scholley, Martina Schaefer, Mark D. Tully, Marc-Andre Hograindleur, Montserrat Soler-López, Roman C. Hillig, Christoph Mueller-Dieckmann and Eaazhisai Kandiah

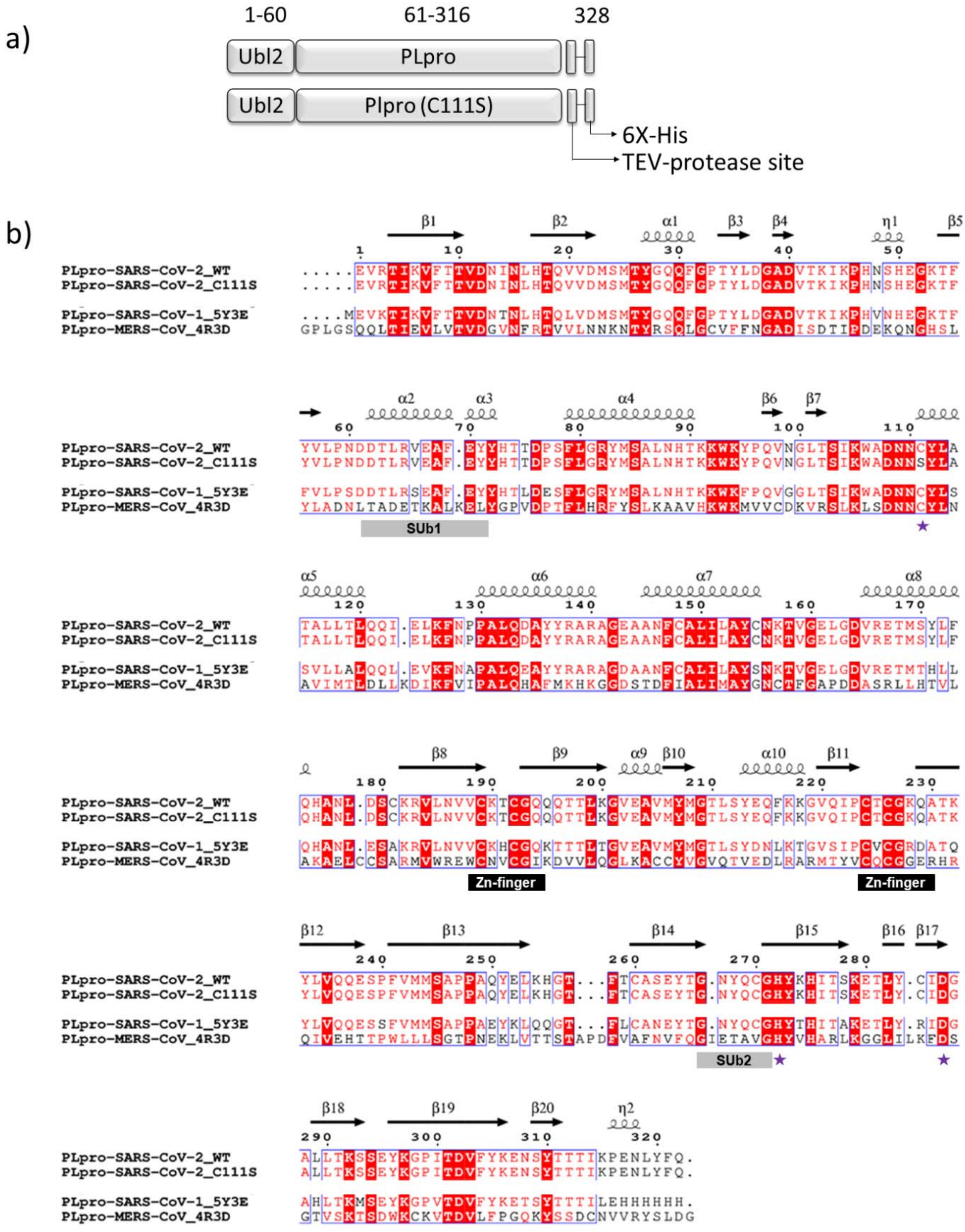

Figure S1 **a)** Domain organisation of the PLPro constructs used in this study **b)** Multiple sequence alignment of PLPro from SARS-CoV-2, SARS-CoV-1 and MERS-CoV. Structures from this study as well as PDB 5Y3E and PDB 4R3D were used for the analysis. Secondary structure elements of PLPro^{WT} from SARS-CoV-2, as identified by ESPRIT (Robert *et al.*, 2025) are shown above the

sequences. The η symbol indicates a 3_{10}-helix. α-helices and 3_{10}-helices are shown as squiggles and β-strands are shown as arrows. Key structural features, including the Zn-finger residues (black boxes), substrate binding sites including SUb1 and SUb2 (grey boxes) and active site residues (purple stars) are indicated below the sequences.

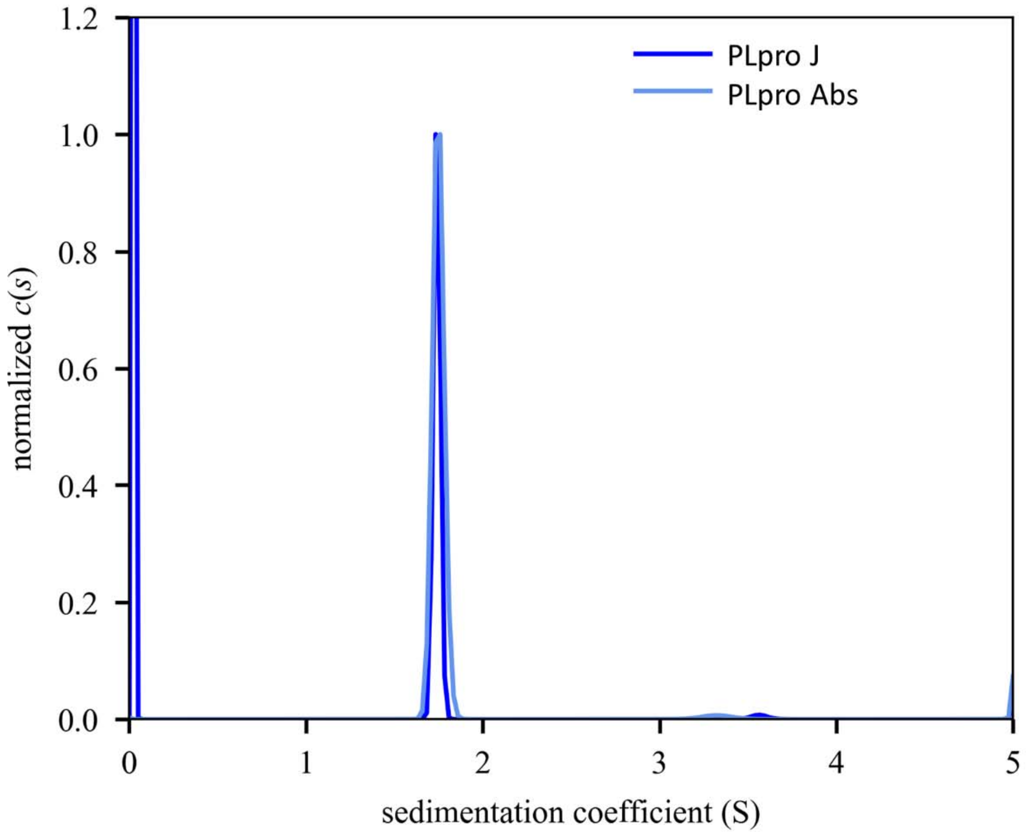

Figure S2 Analytical ultracentrifugation (AUC) results for $PLpro^{WT}$, with a primary peak at 1.73 ± 0.02 S, representing 98 ± 1% of the total. The Non-Interacting Species (NIS) analysis estimates the molecular weight (Mw) at 34.7 ± 2.3 kDa, corresponding to a protomer (theoretical Mw = 35.7 kDa, f/fmin = 1.36–1.38). A minor peak at 3.44 ± 0.11 S accounts for less than 2% of the signal, with NIS analysis giving an Mw of 70.2 ± 2.5 kDa, potentially corresponding to a dimer (theoretical Mw = 71.4 kDa, f/fmin = 1.11)

**Ubl2 residues of protomer B**	**Symmetry related interactions**	**Distance**	**Symmetry**
Glu 1 N	Ser 293 OG	3.38	X,Y,Z+1
Glu 1 OE1	HOH 311 O	3.41	X,Y,Z+1
Glu 1 OE2	HOH 79 O	3.34	X,Y,Z+1
Glu 1 OE2	Lys 297 O	2.76	X,Y,Z+1
Val 2 N	Glu 295 O	3.29	X,Y,Z+1
Val 2 O	Glu 295 N	3.18	X,Y,Z+1
Val 2 O	Glu 295 O	3.43	X,Y,Z+1
Arg 3 NH1	HOH 678 O	2.98	X,Y,Z+1
Arg 3 NH1	Ser 294 N	2.86	X,Y,Z+1
Thr 4 OG1	Ser 294 OG	2.52	X,Y,Z+1

Figure S3 Table showing the symmetry-related interactions of Ubl2 residues of protomer B.

a)

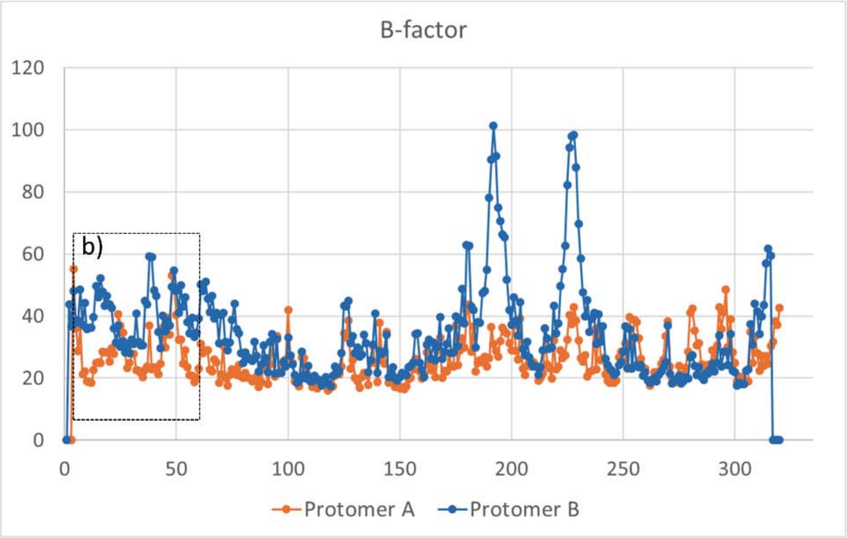

b)

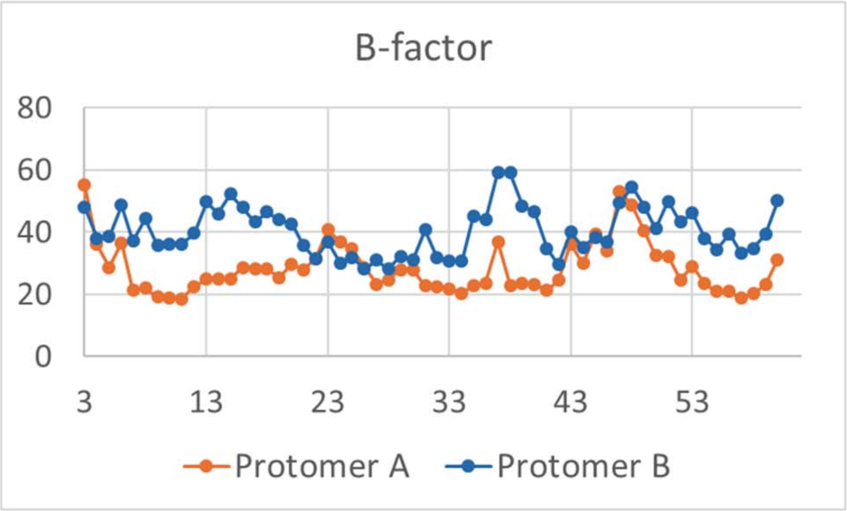

Figure S4 **a)** B-factor distribution for the entire $PLpro^{WT}$ of protomer A and protomer B. **b)** Close-up view of B-factor distribution for the Ubl2 domain (residues 1-60).

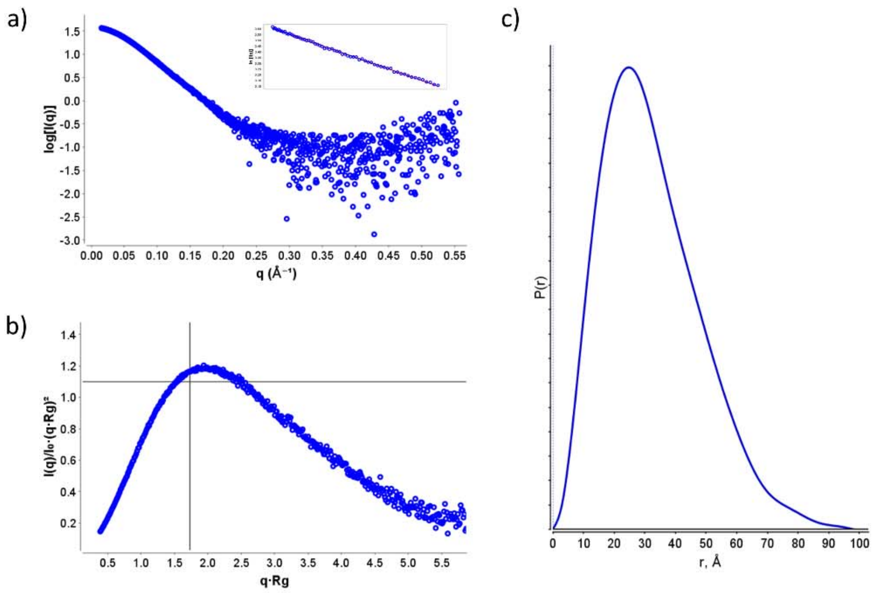

d)

Data-collection parameters	
Instrument:	ESRF BM29
Wavelength (Å)	0.99
q-range ($Å^{-1}$)	0.007-0.5
Sample-to-detector distance (m)	2.81
Concentration (mg/mL)	10
Temperature (K)	293
Detector	Pilatus P3-2M
Flux (photons/s)	$1.4*10^{12}/1*10^{13}$
Beam size at sample (μm)	500*200

Structural parameters	Plpro
I0 (kDa) [from Guinier]	$3.86 x10^{-1}$ +/- $6.1x10^{-2}$
Rg (Å) [from Guinier]	24.98 +/- 0.19
Rg (Å) [from P(r)]	25.4
qminRg - qmaxRg used for Guinier	0.0155 - 0.0521
Volume ($Å^3$)	56609
D_{max} (Å)	98
Calculated theoretical MW (kDa):	36.6
Calculated MW (kDa):	33

software employed	
Primary data reduction:	FreeSAXS, BM29 autoprocessing pipeline
Data processing	ScatterIV

Figure S5 SEC-SAXS of PLPro^{C111S}: **a)** Log_{10} scattering intensity I(q) as a function of the momentum transfer q ($Å^{-1}$). The positive scattering data within the selected q-range are shown. The inset displays the Guinier region (low-q), used to determine the radius of gyration (Rg) under the Guinier approximation, indicating the absence of aggregation and good data quality. **b)** Normalized Kratky plot ($(q·Rg)^2·I(q)/I(0)$ vs q·Rg) used to assess particle flexibility and compactness. The profile is consistent with a predominantly folded, compact particle. **c)** Pair-distance distribution function P(r),

providing real-space structural information. The maximum particle dimension (D_{max}) corresponds to the distance at which P(r) approaches zero while maintaining a smooth and physically meaningful distribution. **d)** Table showing the data collection and structural parameters of the SEC-SAXS experiment of $PLPro^{C111S}$.

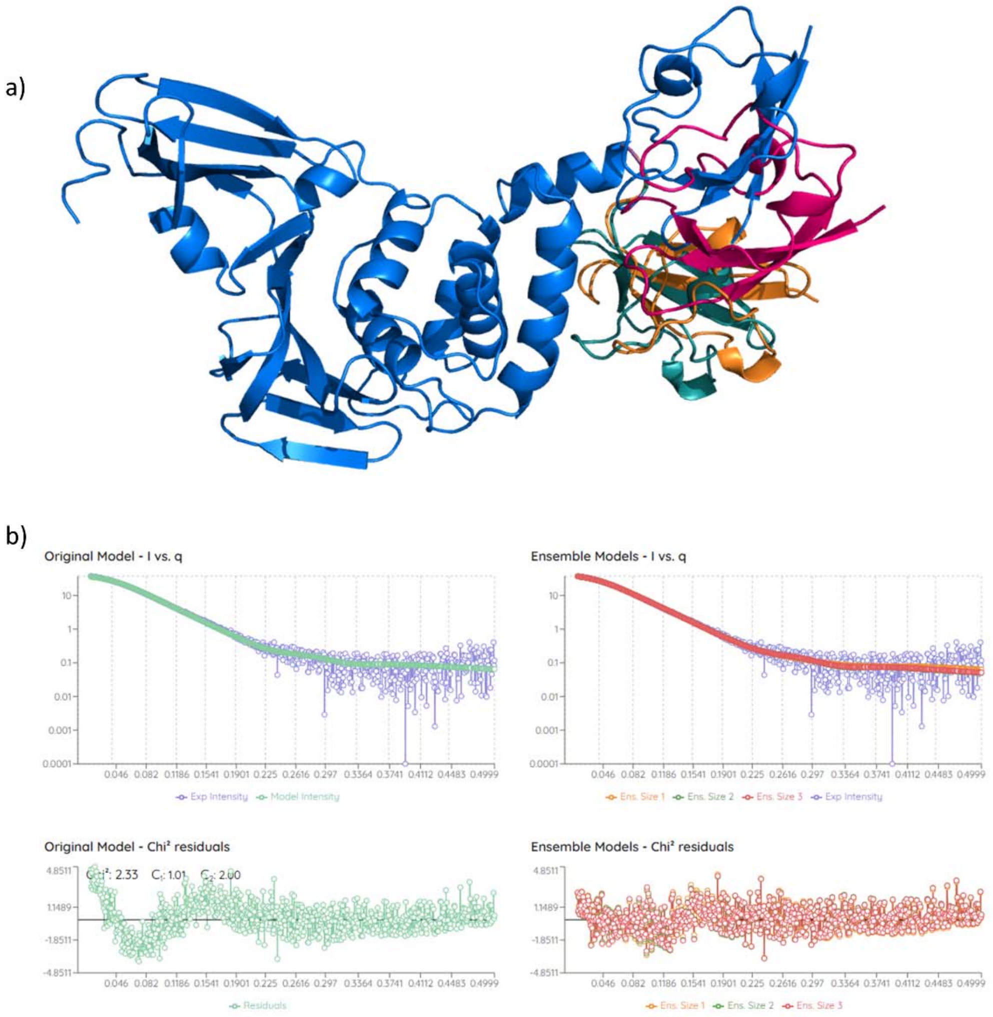

Figure S6 **a)** Ensemble model of PLPro^{C111S} formed using BilboMD showing the possible positions of three models of the Ubl2 domain. **b)** Original model and ensemble model fits to the experimental SAXS data and plots of the residuals.

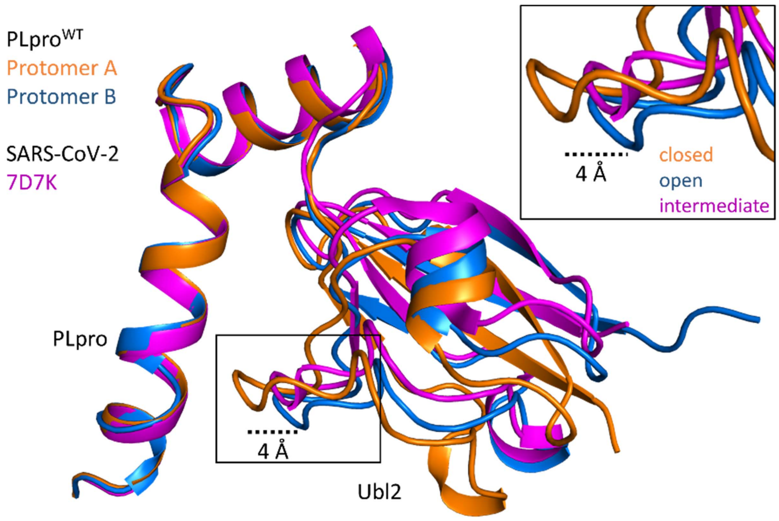

Figure S7 Superimposition of PLpro^{WT} Protomer A and B with 7D7K showing an intermediate conformation in between the open and closed state. An inlet on the top right highlights this intermediate state.

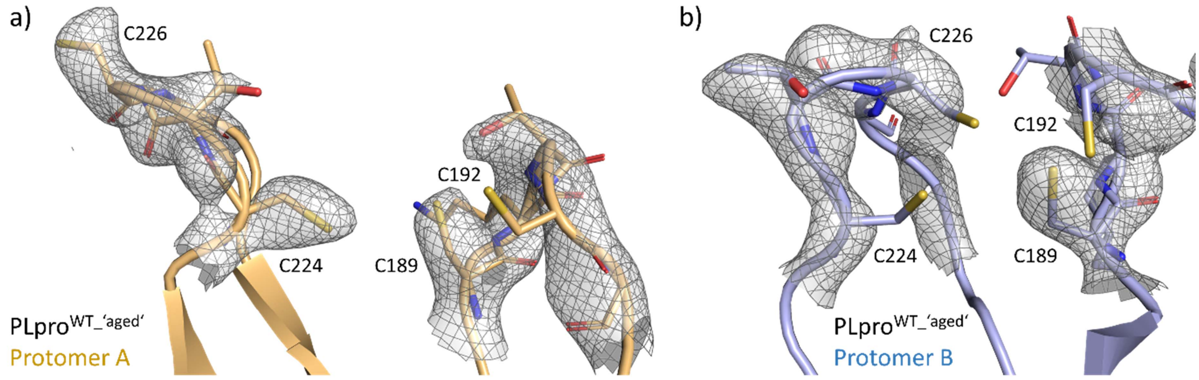

Figure S8 Characterisation of Zn-binding to the Zn-finger domain in the PLpro$^{WT_'aged'}$ crystal structures. Shown here are the Zn-finger residues with the corresponding electron density at a contour level of 1 rmsd for a) protomer A and b) protomer B, showing unresolved sidechains for the Zn-finger residues.

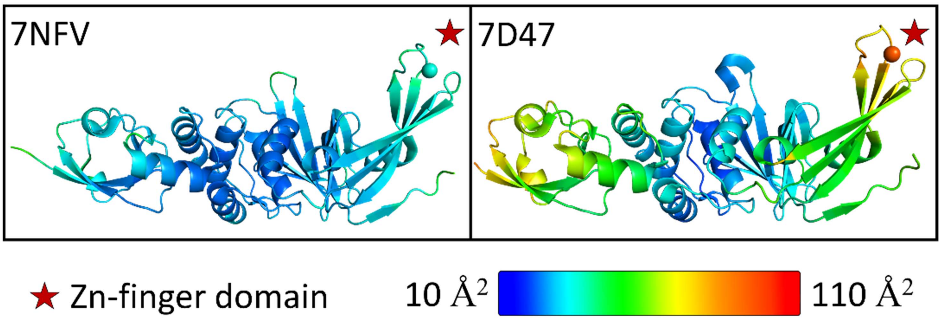

Figure S9 Representation of B-factors in rainbow colour spectrum (ranging from 10 Å^2 to 110 Å^2) of 7NFV (SARS-CoV-2 PLpro) and 7D47 (SARS-CoV-2 PLpro C111S). Higher B-factors can be observed for the Zn finger and Ubl2 regions.

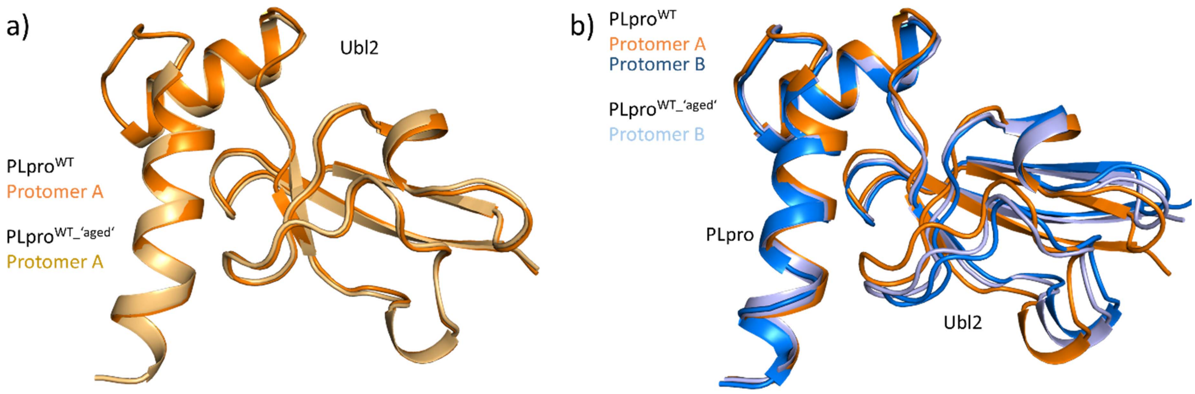

Figure S10 Structural superposition of Ubl2 domain from SARS-CoV-2 PLpro^{WT} and PLpro$^{WT_'aged'}$ crystal structures. For the structure from fresh crystals, protomer A is in orange and protomer B is in blue. For the structure from aged crystal, protomer A is in light orange and protomer B is in light blue. **a)** shows the two Protomer A superimposed with no difference between the two, while **b)** shows PLpro^{WT} Protomer A and B together with PLpro$^{WT_'aged'}$ Protomer B adapting an intermediate conformation.

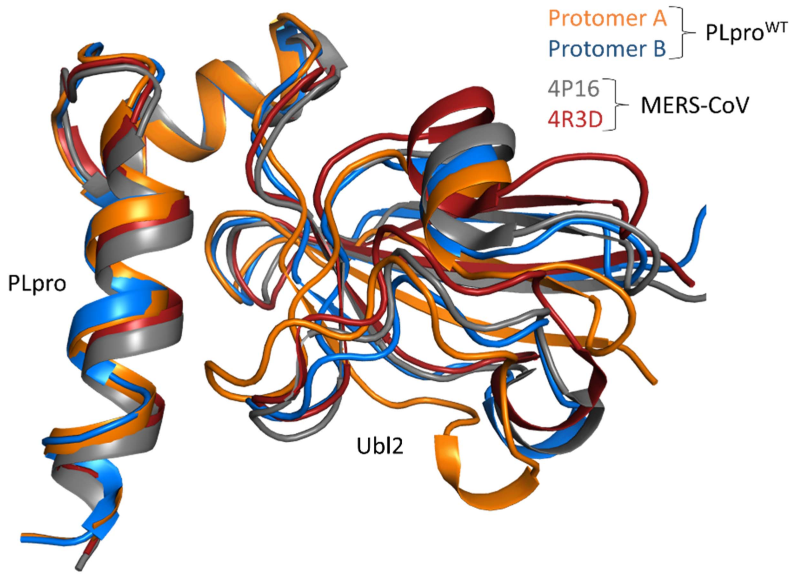

Figure S11 Structural superposition of Ubl2 domain of SARS-CoV-2 PLpro^{WT} structure to various MERS-CoV PLpro structures. For the SARS-CoV-2 PLpro^{WT}, protomer A is in orange and protomer B in blue. For the MERS-CoV PLpro structures, PDB 4P16 (conformation almost identical to 4RNA and 4PT5) is in grey and PDB 4R3D (conformation almost identical to 4REZ) is in red.
